# Phase-preserving beam expander for biomedical X-ray imaging

**DOI:** 10.1107/S1600577515004695

**Published:** 2015-04-15

**Authors:** Mercedes Martinson, Nazanin Samadi, Bassey Bassey, Ariel Gomez, Dean Chapman

**Affiliations:** a Physics and Engineering Physics, University of Saskatchewan, 116 Science Place, Rm 163, Saskatoon, Saskatchewan, Canada S7N 5E2; b Biomedical Engineering, University of Saskatchewan, 107 Wiggins Road, Saskatoon, Saskatchewan, Canada S7N 5E5; c Brockhouse Beamlines, Canadian Light Source, 44 Innovation Boulevard, Saskatoon, Saskatchewan, Canada S7N 2V3; d Anatomy and Cell Biology, University of Saskatchewan, 107 Wiggins Road, Saskatoon, Saskatchewan, Canada S7N 5E5

**Keywords:** beam expander, bent Laue diffraction, double-crystal monochromator, biomedical imaging, dynamic imaging, in-line phase imaging, polychromatic focus

## Abstract

The BioMedical Imaging and Therapy beamlines at the Canadian Light Source are used by many researchers to capture phase-based imaging data. These experiments have so far been limited by the small vertical beam size, requiring vertical scanning of biological samples in order to image their full vertical extent. Previous work has been carried out to develop a bent Laue beam-expanding monochromator for use at these beamlines. However, the first attempts exhibited significant distortion in the diffraction plane, increasing the beam divergence and eliminating the usefulness of the monochromator for phase-related imaging techniques. Recent work has been carried out to more carefully match the polychromatic and geometric focal lengths in a so-called ‘magic condition’ that preserves the divergence of the beam and enables full-field phase-based imaging techniques. The new experimental parameters, namely asymmetry and Bragg angles, were evaluated by analysing knife-edge and in-line phase images to determine the effect on beam divergence in both vertical and horizontal directions, using the flat Bragg double-crystal monochromator at the beamline as a baseline. The results show that by using the magic condition, the difference between the two monochromator types is less than 10% in the diffraction plane. Phase fringes visible in test images of a biological sample demonstrate that this difference is small enough to enable in-line phase imaging, despite operating at a sub-optimal energy for the wafer and asymmetry angle that was used.

## Abstract 2

Building on previous work, a phase-preserving bent Laue beam-expanding monochromator was developed with the capability of performing live animal phase contrast dynamic imaging at the Biomedical Imaging and Therapy beamline at the Canadian Light Source.

## 1. Introduction   

At the Canadian Light Source (CLS) in Canada, the BioMedical Imaging and Therapy (BMIT) bend magnet (BMIT-BM) beamlines and insertion device (BMIT-ID) (Wysokinski *et al.*, 2007 ▶, 2013 ▶) have been very successful in their mission to image biological tissue and conduct live animal imaging studies (Pratt *et al.*, 2014 ▶). However, since their inception, they have been limited by the vertical beam size. This poses limitations for imaging modalities such as micro-computed tomography and dynamic phase imaging, techniques which are necessary to remain at the cutting edge of biomedical imaging research.

Previous results (Martinson *et al.*, 2014 ▶) reported a vertical beam expansion of approximately 7.7×. During these experiments, we discovered that the beam expander destroyed the phase characteristics of the beam in the vertical direction (corresponding to horizontal edges in the object) and caused blurring of horizontal (but not vertical) knife-edges placed at longer sample-to-detector distances. We have taken a two-pronged approach to solving this problem. On the theoretical side, we derived a better approximation for the polychromatic focal length, allowing us to carefully merge it with the well established geometric focus equation. At the same time, we developed a bending frame that allowed us to more carefully control the bend radius of the crystal. The result of this effort is a great improvement in the coherence of the expanded beam, enabling techniques such as dynamic phase imaging at the BMIT beamlines.

## 2. Background theory   

**Figure 1:**
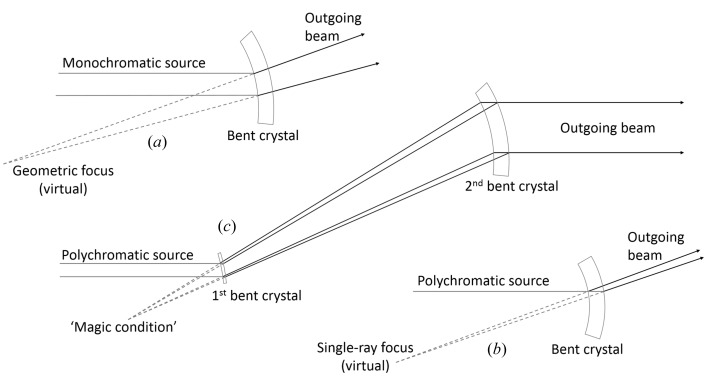
(*a*) Geometric (virtual) focus of two incoming monochromatic rays by a bent Laue crystal. (*b*) The single-ray (virtual) focus of a polychromatic beam by a bent Laue crystal. (*c*) The two crystals aligned such that the single-ray and geometric foci of the first crystal coincide with each other and with the geometric focus of the second crystal. The first crystal satisfies the ‘magic condition’.

In order to observe edge-enhancement refraction effects, the source must be angularly small (either physically small and/or far away), which imparts a high degree of transverse coherence to the wavefronts, hereto referred as phase coherence or simply coherence. Apparent angular source size is adversely affected when the two types of bent Laue crystals focus – single-ray (polychromatic) and geometric (monochromatic) – are mismatched. In order to preserve the beam coherence, these two focal points must coincide (Suortti *et al.*, 1993 ▶) in what is referred to here as the ‘magic condition’ (Fig. 1 ▶). The magic condition restores the source to its proper angular size, thus preserving the edge-enhancement effects referred to here as the phase properties of the images.

Given a crystal with a specified asymmetry angle, the Bragg angle can be chosen so that the two focal points coincide. However, this determines the energy used for the experiment, so an alternative method is to first choose a convenient energy and reflection (preferably with high reflectivity to maximize flux), and then choose the corresponding asymmetry angle accordingly. Unfortunately, obtaining custom-cut crystals with uncommon asymmetry angles is often at a prohibitive cost. A compromise is to use readily available off-cut crystals with asymmetry angles close to ideal, and then to allow some variance in the Bragg angle.

**Figure 2:**
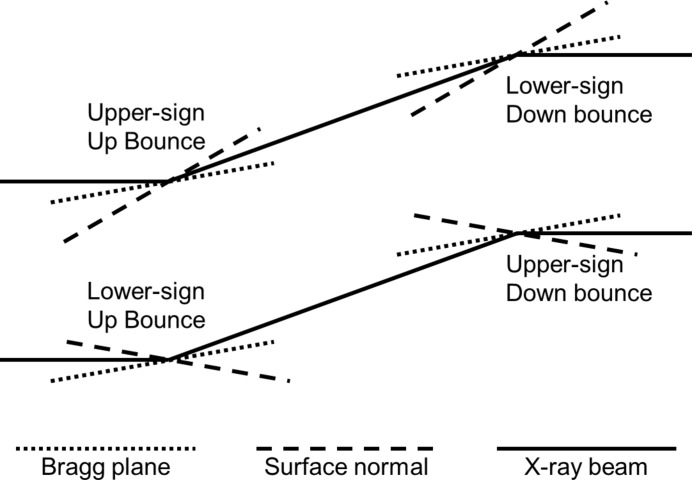
Upper-sign and lower-sign geometries.

Both geometric and single-ray focal lengths are a function of the Bragg angle θ_B_, the asymmetry angle χ and the crystal bending radius *R*. The geometric focus *f*_g_ is also a function of the source-to-crystal distance *f*_s_ and is given implicitly by (Schulze *et al.*, 1998 ▶)
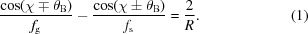
The usual sign convention is used, where the focus is real (virtual) if 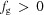 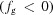. Assuming the source is far away 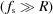, then *f*_g_ and *R* have the same sign. This motivates a sign convention for the bend radius such that 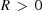 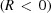 when the source is on the convex (concave) side of the crystal. The upper/lower-sign convention refers to the angle 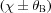 between the incoming beam and the surface normal, as shown in Fig. 2 ▶.

**Figure 3:**
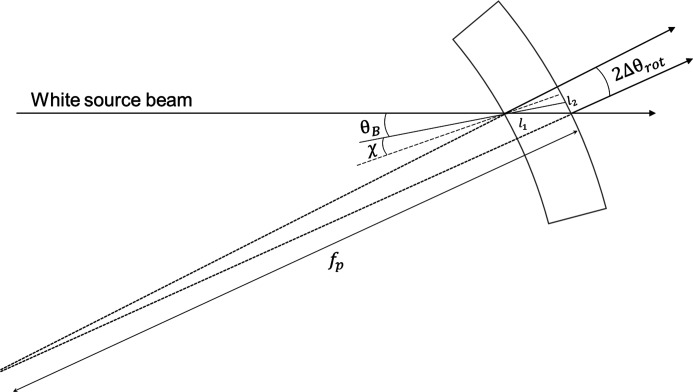
Polychromatic focus diagram.

At a first approximation, the polychromatic focus has been previously given (Suortti *et al.*, 1993 ▶) as
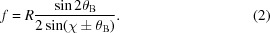
However, this does not account for the curvature of the diffraction planes induced by the elastic deformity of the crystal. For a better approximation, consider a crystal with Poisson ratio ν and thickness *T*. We examine the path of the pencil beam through the crystal to determine the angle between a ray diffracted at the incident surface and a ray diffracted at the exit surface (Fig. 3 ▶). As an incident ray traverses the crystal, it sees a change in Bragg angle (Erola *et al.*, 1990 ▶) of 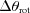:
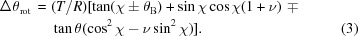
This form of the equation is useful for measuring the bandwidth of a diffracted pencil beam; however, it requires modification for use in the derivation of the polychromatic focal equation. First, it provides only the magnitude of the change in Bragg angle, not the sign. This is important because a pencil beam will create either a virtual or real focus depending on the upper- or lower-sign geometry as well as the orientation of the crystal concavity relative to the source. In keeping with our earlier sign convention for the bend radius, we modify the equation by multiplying by (−1) when we are in lower-sign geometry. The second modification stems from the third term in the equation, 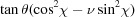, which accounts for the change in *d*-spacing as the beam passes through the crystal. While this affects the energy bandwidth of the exiting beam, it does not change the focus properties, and so is dropped from our modified equation. The final form is then
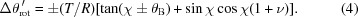
The path length of the non-diffracted ray through the crystal is 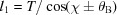. At the exit surface, the diffracted rays compose a beam with width 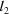 = 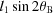 = 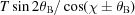. Using the small-angle approximation, 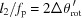, and solving for the polychromatic focus, we have
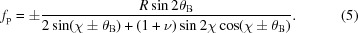
This agrees with a previous result (Sutter *et al.*, 2008 ▶) that was derived for a bent Laue crystal in the lower-sign orientation. Their derivation appears to use the convention that 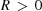, and instead brings the negative sign into the equation for the case where the source is on the concave side of the crystal.

## 3. Design and implementation   

Physical constraints of the imaging hutch require small Bragg and asymmetry angles. For this experiment, we had ready access to a crystal-reflection pair meeting this requirement, namely a (5,1,1) crystal wafer (5′′ diameter, high-resistivity FZ) with (3,1,1)-type reflection and an asymmetry angle of 3.33°. The magic condition is found by numerically solving *f*_p_ = *f*_g_ for the Bragg angle. Using χ = 3.33°, ν = 0.22, *f*_s_ = 22 m and bend radius *R* = −0.5 m, we find that the magic condition is met for upper-sign geometry with θ_B_ = 7.55°. The lower-sign geometry yields a numerical solution of θ_B_ = 352.45°, which is physically the same as θ_B_ = 7.55° in upper-sign geometry, and so is discarded. Returning the expanded beam to the horizontal direction requires that the planes of the second crystal be aligned with those of the first. This plane matching puts the second crystal in lower-sign geometry, making it impossible to preserve the magic condition through both crystals.

In order to meet the physical constraints of the rail system installed in the BMIT-BM beamline hutch, we tolerated a slight deviation from the magic condition, specifically a Bragg angle of 6.56°. From previous work (Zhu *et al.*, 2014 ▶), we knew that this Bragg angle adequately approximates the magic condition and, furthermore, corresponds to the *K*-edge of iodine, allowing us to confirm the energy. Ideally we would have preferred to choose this energy first and then obtain custom-cut crystals to match, but this was not feasible for this experiment.

**Figure 4:**
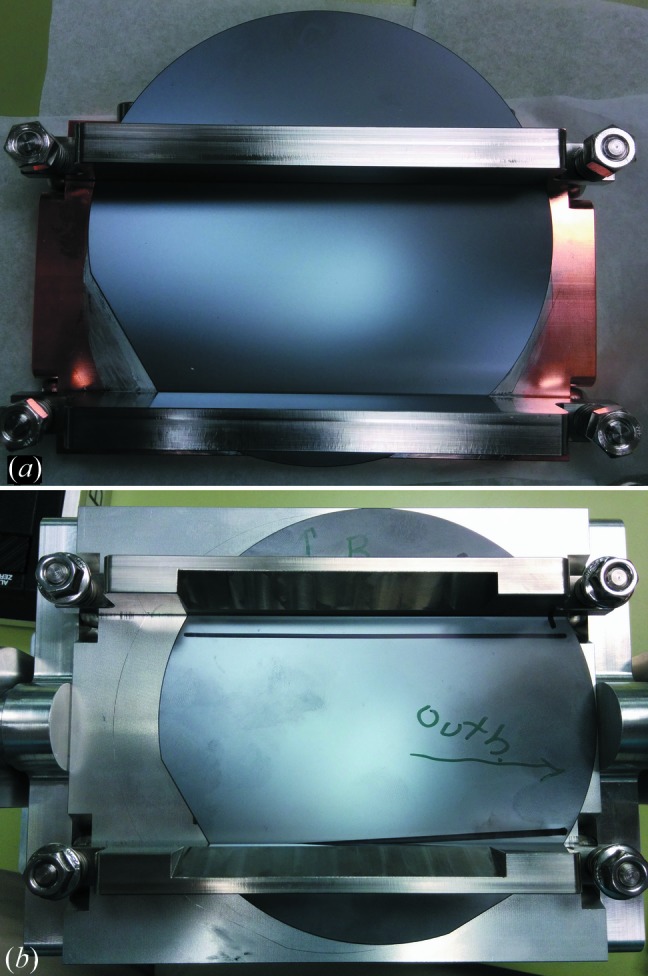
(*a*) First crystal (vacuum-compatible) and (*b*) second crystal mounted on rigid frame benders. In each figure, the wafers are 5′′ diameter. As shown, the beam would be coming out of the page.

Besides merging the geometric and single-ray foci, the beam-expanding monochromator was improved through the development of rigid frame benders (Fig. 4 ▶). The main advantage of a frame bender is the fixed bend radius. The four-bar bender previously used allowed too much variation between experiments and introduced uncertainty as to the true bend radius of the crystal. The frame bender improves reproducibility between crystals, allowing us to mount and swap crystals quickly. It was discovered that crystals designed for the semiconductor industry are not machined with a highly precise asymmetry angle, leading to frequent mismatch between first and second crystal. The simplest way to overcome this variation is by trial-and-error matching in the beamline, which would never have been practical with the lengthy process required for mounting crystals in the four-bar bender. As a solid metal object, the frame bender also supports heat dissipation and water cooling for the first crystal, which is exposed to the full white beam of the wiggler beamline.

The biggest disadvantage of the frame bender is that it requires extremely high-precision machining, which is not readily available at the CLS. Surface irregularities on the order of 10 µm seem sufficient to distort the crystal shape enough to cause mismatch between the crystals and ultimately loss of intensity and phase characteristics. Because the first bender must be machined in solid copper for optimal heat dissipation, the additional cost renders this level of high-precision machining unfeasible. Attempts to smooth the surface either by additional machining or by inserting smooth intermediate layers distorted the bend radius and triggered catastrophic loss of intensity.

**Figure 5:**
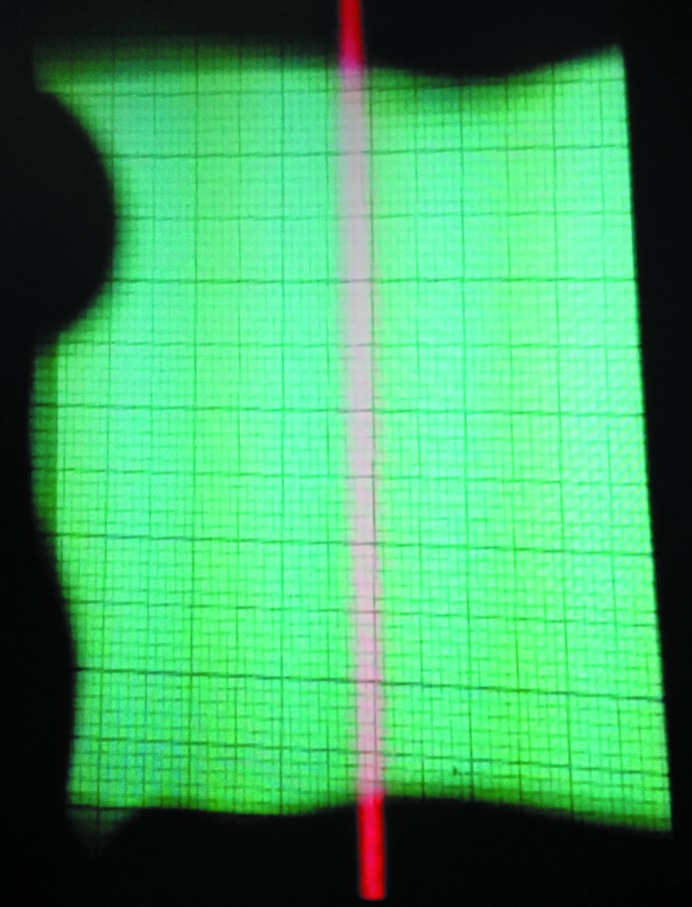
Expanded beam at BMIT-ID beamline on fluorescent paper with cm-scale major grid lines.

Despite the challenges in machining, the wide rocking curve of the severely bent crystals allowed us to achieve an expansion of approximately 10× with adequate uniformity on the BMIT-ID beamline (Fig. 5 ▶).

## 4. Image analysis and results   

By matching the polychromatic (single-ray) focus to the geometric (multiple ray) focus, we were able to preserve the phase coherence of the X-ray beam while expanding its vertical size by a factor of 12 on BMIT-BM. The final beam size measured 50 mm (V) × 70 mm (H), the exact size of the window in the frame bender of the second crystal, leading us to believe that the true expansion may actually be greater.

**Figure 6:**
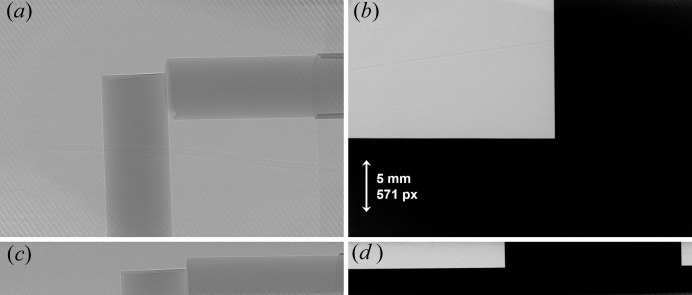
Flat-corrected phase and knife-edge images used for analysis, also demonstrates field of view of respective monochromators. (*a*, *b*) Bent Laue monochromator, (*c*, *d*) flat Bragg monochromator, (*a*, *c*) lucite rods, (*b*, *d*) tungsten block. Propagation distance is 134 cm. All images have the same scale.

A visual inspection of in-line phase images (Fig. 6 ▶) of a strong phase producing test object (Lucite rods) and a knife-edge (tungsten carbide block) showed excellent phase properties in both the vertical and horizontal directions, demonstrating preservation of beam divergence through the expander. The test objects were imaged with a sample-to-detector propagation distance of 134 cm. These images, acquired using both the expander and the BMIT-BM beamline monochromator, were analysed and compared.

**Figure 7:**
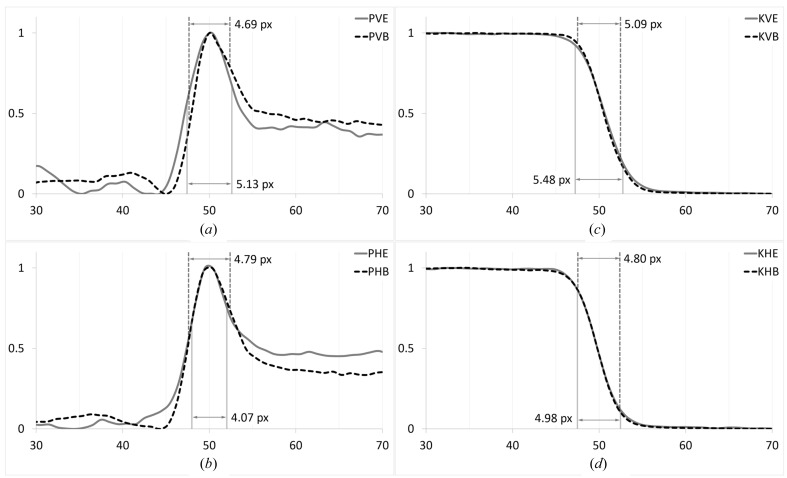
Plot profiles with relative intensity on the *y* axis and pixel range on the *x* axis. Image type: [K]nife edge or [P]hase fringe. Edge normal: [V]ertical or [H]orizontal. Monochromator type: [B]eamline or [E]xpander. (*a*) PVE and PVB, (*b*) PHE and PHB, (*c*) KVE and KVB, (*d*) KHE and KHB.

The phase images were analysed using an asymmetric pseudo-Gaussian measurement technique. The peak width across a phase fringe was measured as the distance (in pixels) between the inflection points of the increasing and decreasing sides of the plot profile (Figs. 7 ▶ *a* and 7*b*). The knife-edge images were analysed by fitting the derivative of the plot profile (Figs. 7 ▶ *c* and 7*d*) of the knife-edge to a Gaussian and measuring the peak width as FWHM. Peak width results for each data set are reported in Table 1 ▶, along with the percent difference between monochromator type. Positive (negative) differences indicate that the expanding monochromator causes more (less) degradation than the beamline monochromator. From these results, it is evident that the beam is more strongly affected in the vertical direction, although there are also some horizontal effects likely caused by anticlastic bending. We expect the beam to be degraded in the diffraction plane (vertical direction), but we see that this effect is less than 10%. In the horizontal plane, the difference is less than 15%. Because the incident beam is produced by a synchrotron, the coherence of the expanded beam is still well within tolerance for phase-based imaging techniques.

**Figure 8:**
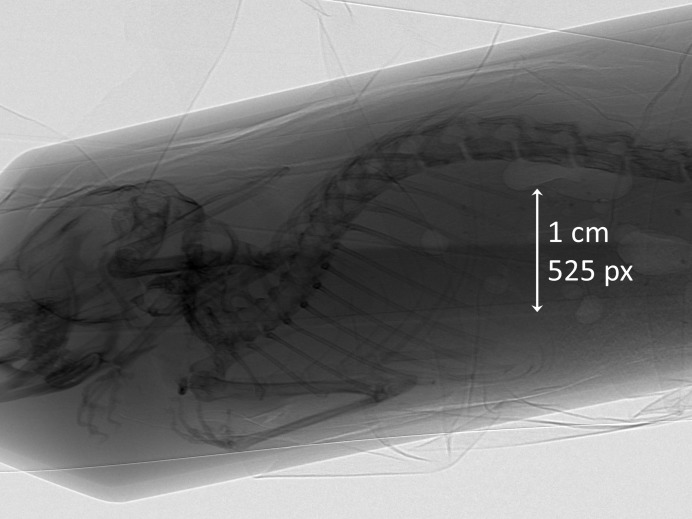
Phase image of euthanized mouse. Propagation distance approximately 200 cm .

Additional imaging tests were done on biological samples (euthanized mice acquired from other experiments), demonstrating the true capability of the biomedical imaging system (Fig. 8 ▶).

There are other potential applications for this crystal geometry using the magic condition that preserves phase. One common application of dual bent Laue crystals is microfocusing synchrotron beams. Using this particular geometry and magic condition, it should be possible to create these microbeams in a way that preserves beam coherence and phase properties. This could have applications to microprobe and crystallography.

## 5. Conclusion   

We have demonstrated that a beam-expanding monochromator is able to preserve the beam coherence and phase properties of a synchrotron beam by carefully matching the geometric and polychromatic focal lengths of two cylindrically bent crystals in Laue diffraction mode. Imaging experiments demonstrated excellent phase contrast in a biological sample as well as phase and knife-edge test objects. Visual analysis confirmed that horizontal and vertical phase fringes and knife-edges were comparably affected, and the numerical analysis confirmed that the differences between them were less than 10% in the diffraction (vertical) plane. While this is not perfect, it is noted that we were not operating at the optimal Bragg angle on account of the apparatus already in place at the beamline. Future work will repeat the phase and knife-edge experiments at the exact energy determined by numerically solving the equations.

While these experiments were done in ‘expansion mode’, it should be possible to reverse the crystals and run the system in ‘compression mode’. Everything discussed in this work should hold true in the reverse orientation, as only the sign of the bend radius will change. This modality will be the topic of future work.

## Figures and Tables

**Table 1:** Summary of analysis results

Image type	Edge normal	Monochromator	Peak width (pixels)	Percent difference
Phase fringe	Vertical	Expander	5.13	9.4
		Beamline	4.69	
	Horizontal	Expander	4.07	15
		Beamline	4.79	
Knife-edge	Vertical	Expander	5.48	7.7
		Beamline	5.09	
	Horizontal	Expander	4.98	3.8
		Beamline	4.80	
